# The emergence of Beijing genotype of *Mycobacterium tuberculosis* in the Kingdom of Saudi Arabia

**DOI:** 10.4103/1817-1737.65045

**Published:** 2010

**Authors:** Sahal Al Hajoj, Nalin Rastogi

**Affiliations:** *Department of Comparative Medicine, King Faisal Specialist Hospital and Research Centre (MBC 03), PO Box 3354, Riyadh - 11211, Saudi Arabia*; 1 *Pasteur Institute of Guadeloupe, Tuberculosis and Mycobacteria Unit, BP 484, F97183-Abymes, Cedex, Guadeloupe, (France)*

**Keywords:** Beijing genotype, emergence, *Mycobacterium tuberculosis*, Saudi Arabia

## Abstract

**OBJECTIVE:**

To investigate the prevalence of Beijing genotype strains of *Mycobacterium tuberculosis* in the Kingdom of Saudi Arabia.

**METHODS:**

We analyzed the available data on a total of 1505 strains isolated during 2002–2005.

**RESULTS:**

Spoligotyping results revealed that Beijing family isolates represented 4.5% of all the isolates. Existence of Beijing clade is alarming as this family is known to be multi-drug resistant and transmissible.

**CONCLUSIONS:**

This study showed that the occurrence of Beijing genotype is associated with young age and drug resistance. The Beijing strains affected both Saudi nationals as well as migrants originating in Asia. The Beijing clade could be responsible for the ongoing transmission of tuberculosis within the community.

With 8 million new cases and 2 million deaths every year, tuberculosis (TB) remains one of the major causes of public health problems worldwide. According to the World Health Organization (WHO), due to its interaction with the human immunodeficiency virus (HIV) epidemic cases is likely to increase in many areas of the world. There is also an emergence of multidrug-resistant tuberculosis (MDR-TB) strains mainly due to the poorly managed TB control programs. However, according to the fourth report on anti-TB drug resistance in the world, this information is only scarcely available for WHO Eastern Mediterranean region that includes Saudi Arabia. Current TB drug-resistance trends are available only for the gulf states of Oman and Qatar, both with small numbers of total cases and low-to-moderate levels of resistance, most of which is imported. However, estimates of MDR-TB in Saudi Arabia among all TB cases (n = 11,024) are projected at 3.4% (i.e., 2.2% of the 10,631 new cases and 36.4% of the 393 previously treated cases).

In the above context, the Beijing lineage strain of *Mycobacterium tuberculosis* has been extensively reported in the literature for its positive association with MDR-TB and major outbreaks.[1,2] Initially described in 1995 as a genetically closely related group of tubercle bacilli from the People‘s Republic of China, it is a common strain in East Asia, which is hypothesized to expand from a single ancestor with selective advantage.[3] In the past decade, many studies have pointed out the significance of the Beijing strains in the worldwide TB epidemic and emergence of MDR-TB, e.g., in Southeast Asia, former USSR Republic, the Baltic States and South Africa (reviewed in Ref. 4). The wide distribution of the Beijing family in distinct geographic regions and its ability to spread in clonal clusters is suggestive of a recent dissemination and also suggests that the members of this family are better adapted to infect humans.[4] In a study carried out in 6829 *M. tuberculosis* isolates in the Netherlands, a 6% prevalence of the Beijing genotype and an association of the Beijing genotype with nationality, young age and MDR–TB was observed, where the genotype was shown to be predominant among the immigrants from Asia.[5]

No report has yet focused on the distribution of Beijing isolates in Saudi Arabia, despite an escalated risk due to annual pilgrimage to Mecca and Medina. According to the Royal Embassy of Saudi Arabia, the number of foreign pilgrims in Saudi Arabia to perform the “Hajj” rose from 1,080,465 in 1996 to 1,729,841 in 2008. This includes mostly adult (often elderly) pilgrims coming from TB endemic regions, and taking part in a month long overcrowded event. In addition to such a high influx of visitors and pilgrims, Saudi Arabia is a host to approximately 6 million resident foreigners coming from all over the world – mainly from countries with a high incidence of TB, e.g., Arabic-speaking countries (mainly Egypt, Yemen, Jordan, Syria), followed by the Indian subcontinent (India, Pakistan, Bangladesh, Sri Lanka), and the Philippines. A study focusing on the causes of pneumonia during the 1994 pilgrimage (Hajj) season to Mecca reported that all the patients enrolled were from developing countries,[6] among whom TB diagnosis was established in 72% with *M. tuberculosis* being the commonest causative organism (total mortality rate 17%). Consequently, we aimed at investigating the prevalence of Beijing genotype in Saudi Arabia in conjunction with parameters such as age, sex and nationality of the patients, as well as its association with MDR-TB.

## Methods

In this study, we reviewed data from a total of 1505 clinical isolates recovered from as many patients with culture confirmed TB from various regions of the Kingdom, from January 2000 to December 2005 [Table 1]. All the cases were reported to the Ministry of Health by the regional government hospitals, and the demographic, epidemiologic and clinical data available included gender, age, nationality, site of infection, HIV status and history of the disease.[7] All the isolates were identified as *M. tuberculosis* complex using standard bacteriology, culture and biochemical tests, followed by drug-susceptibility testing (DST) using the Bactec MGIT960 system (Becton-Dickinson, Maryland, USA) as per the manufacturer’s recommendations, at the King Faisal Specialist Hospital and Research Centre (KFSHRC). Genomic DNA was extracted using the cetyl-trimethyl-ammonium-bromide method (CTAB), followed by spoligotyping as previously reported.[8] Beijing genotype strains were identified based on the specific spoligotyping signature of this lineage – defined as Spoligotype International Type (SIT) 1 in the international database SpolDB4 of the Pasteur Institute of Guadeloupe,[9] available online at http://www.pasteur-guadeloupe.fr:8081/SITVITDemo. Chi-square test was used to detect the significance of the differences between groups. The type I error rate was set at 5%. *P* value less than 5% was declared as significant.

**Table 1:** Regions and spread of Beijing genotype

City/region	Total no. of isolates	Beijing genotype	%
Riyadh	428	19	3.7
Dammam	419	29	7.0
Taif	443	14	3.3
Medina	117	5	4.2
Jizan	25	0	0
Al-Baha	13	0	0
Tabuk	60	0	0

## Results

The Beijing genotype (SIT1), characterized by the absence of spacers 1–34[10] was identified in 67/1505 or 4.45% of the patients with the following distribution pattern in Saudi Arabia: Riyadh 19/428 (4.4%), Dammam 29/419 (6.9%), Taif 14/443 (3.16%), Medina 5/117 (4.3%), and none in Jizan (*n* = 25), Tabuk (*n* = 60), and Al-Baha (*n* = 13). The patient data available for these 67 Beijing isolates are summarized in Table 1. It shows that the proportion of TB cases with the Beijing genotype was highest among the Saudi nationals (30 cases) and Asians (27 cases). The *P* values were 0.0001 for both parameters when compared to other nationals. This may be an indication of the rising number of imported TB cases through Asian immigrant workers in the country. It also suggests that the methodology for screening guest workers for TB prior to the delivery of the work permit is not effective, although some might have reactivated their primary infection late after arrival. Unfortunately, we could avail neither a record of their duration of stay in the Saudi kingdom prior to the diagnosis for TB infection nor a detailed medical history allowing us to discriminate the cases of newly acquired infection, reinfection, and reactivation. Furthermore, Beijing genotype preferentially infected persons of most productive age groups as compared to the rest of the age groups (21–40 years, *n* = 44/67 or 65.67%; 41–60 years, *n* = 11/67 or 16.42%; *P* = 0.0001) [Table 2]. This is a clear indication of an ongoing transmission as these age groups concern both local citizens and expatriates. Almost a quarter of the Beijing isolates (15/67 or 22.4%) was associated with any drug resistance, among which more than half (9/15) were associated with multidrug resistance [Table 3]. However, no statistically significant association of drug resistance of the Beijing isolates was observed with the parameters studied, i.e., gender, age group, nationality and HIV serology.

**Table 2:** Demographic data for the Beijing genotype of *M. tuberculosis* found in Saudia Arabia

Demographic data	No. of Beijing genotype	%
Year of diagnosis		
2000	3	3.7
2001	1	0
2002	7	9.2
2003	17	26.0
2004	34	53.7
2005	5	5.5
Sex		
Male	34	52.0
Female	33	48.0
Age group (years)		
0–20	8	11.1
21–30	21	31.5
31–40	23	35.2
41–50	8	11.1
51–60	3	1.8
Above 60	4	3.7
Nationality		
Non-Saudi		
Asian	27	40.0
African	2	2.0
Unknown	8	11.1
Saudi	30	46.2
HIV serology		
Positive	0	0
Negative	67	100

**Table 3:** Drug resistance analysis of Beijing genotype with demographic and clinical available data

	MDR (RIF + INH)	STR + 1NH	STR	INH	RIF
Total no. of isolates	9	2	1	1	2
Year of diagnosis					
2000					
2001					
2002		1			1
2003	2	1		1	
2004	7				1
2005			1		
Sex					
Male	4	0	1		1
Female	5	2		1	
Age group					
0–20	2				
21–30	2	2	1	1	1
31–40	3				
41–50	2				1
Nationality					
Non-Saudi					
Asian	5	1			1
African			1		
Saudi	4	1		1	
HIV serology					
Positive					
Negative	9	2	1	1	1

MDR (RIF + INH) = Multidrug Resistance TB for Rifampicin and Izoniazide; SRT = Streptomycin; INH = Izoniazide; STR = Streptomycin

## Discussion

This is the first report on the prevalence of Beijing genotypes, their association with drug resistance, young age, and immigration in Saudi Arabia. Presence of Beijing genotypes is not surprising as the country harbors millions of workers mainly from Southeast Asia, in addition to millions who visit the country for Islamic rituals every year. However, the presence of this genotype in 4.5% of the isolates is alarming, especially considering its association with drug resistance, young age and immigration. Although ongoing transmission among migrant workers, among Saudi nationals, and between the two groups is highly suspected, only a second-line molecular typing using extended 24-loci MIRU-VNTR markers (variable-number tandem-repeats[11]) can give a conclusive evidence. Indeed, the classical Beijing genotype strain (SIT1) shows no genotypic diversity on spoligotyping as it is characterized by the absence of spacers 1–34 upon spoligotyping.[10] Hence, the need arises to subject these strains to VNTR typing using 24 loci of a newly proposed format and three hypervariable (HV) loci namely, QUB-3232, VNTR-3820, and VNTR-4120.[12]

The association of the Beijing genotype with multidrug resistance, young age, and recent diagnosis has to be studied in detail in Saudi Arabia covering the period 2006–2009 to confirm the present trend of almost a quarter of the strains as being drug-resistant (one-eighth being MDR-TB). Indeed, not all strains of Beijing family are associated with drug resistance. Based on the data available on >29,000 patients from 49 studies in 35 countries.[1] It showed that reportedly four patterns exist for Beijing isolates worldwide: (i) endemic, not associated with drug resistance (high level in most of East Asia, lower level in parts of the United States); (ii) epidemic, associated with drug resistance (high level in Cuba, the former Soviet Union, Vietnam, and South Africa, lower level in parts of Western Europe); (iii) epidemic but drug sensitive (Malawi, Argentina); and (iv) very low level or absent (parts of Europe, Africa). Even though the extent of the prevalence of Beijing genotypes is not yet precisely established for the whole of Saudi Arabia (see subsequently), our study confirms that Beijing genotype TB is an emerging pathogen in several areas of the country and it is already being transmitted as an endemic strain among some Saudi nationals. It is also frequently associated with drug resistance, underlining that first two patterns reported by the European Concerted Action[1] already exist in Saudi Arabia.

Nonetheless, one of the drawbacks of our study is the fact that no isolates were collected from Mecca which is the biggest holy city in Saudi Arabia and harbors more than 2 millions pilgrims at the same time and same place. Furthermore, recruitment was not optimal for many provinces due to a lack of a properly functioning set-up of the TB centre, e.g., Beijing genotype corresponded to only 5/117 isolates (4.3%) in Medina and none in Jizan (*n* = 0/25), Tabuk (*n* = 0/60), and Al-Baha (*n* = 0/13). Small number of isolates or no isolates at all may have an impact on our results as one may not rule out the presence of Beijing genotype in these regions. Despite this drawback, our study is the first to underline ongoing transmission with the notorious Beijing strain in Saudi Arabia. Beijing genotype commonly found in Asian countries has today spread worldwide.[1–3,5] Nonetheless, its prevalence and its association with drug resistance have only been scarcely studied in the Middle East, with the exception of Iran, showing an overall prevalence of Beijing genotype of 3.2%,[13] and a proportion of 21.7% of the isolates being associated with multidrug resistance.[14] There is also an increasing evidence of intra-community transmissions between Iranian and Afghan patients among clustered cases (including MDR-TB clusters), with a significant proportion of Beijing isolates.[15] In this context, it is worth mentioning that in addition to considerable numbers of Afghani workers scattered all over Saudi Arabia, both Iranians and Afghanis are among the major Muslim pilgrims to Mecca and Medina. Asian migrant workers (mainly from the Philippines, Bangladesh, and Pakistan) also form considerable societies in the country. Consequently, the possibility that Beijing genotype TB cases are imported in the country by Afghanis, Iranians, Southeast Asians, or other nationals with known Beijing cases,[1–3,13–15] is far from negligible.

In conclusion, Saudi Arabia harbors Beijing genotype (4.5% of all isolates), which is associated with drug resistance and younger age of the patients. Association of multidrug resistant Beijing strains with young age group might be an indication of an ongoing transmission and subsequent spread of multidrug resistance in the country. Further studies covering the whole country for additional time period (2006–2009), and second-line molecular typing using MIRU-VNTR markers are urgently required to reveal the real impact of Beijing genotype on prevailing tuberculosis epidemiology and *M. tuberculosis* drug resistance in Saudi Arabia.

## References

[1] (2006). European Concerted Action on New Generation Genetic Markers and Techniques for the Epidemiology and Control of Tuberculosis. Beijing/W genotype *Mycobacterium tuberculosis* and drug resistance. Emerg Infect Dis.

[2] Glynn JR, Whiteley J, Bifani PJ, Kremer K, van Soolingen D (2002). Worldwide occurrence of Beijing/W strains of *Mycobacterium tuberculosis*: A systematic review. Emerg Infect Dis.

[3] van Soolingen D, Qian L, de Haas PE, Douglas JT, Traore H, Portaels F (1995). Predominance of a single genotype of *Mycobacterium tuberculosis* in countries of East Asia. J Clin Microbiol.

[4] van Soolingen, D, Kremer K (2009). Findings and ongoing research in the molecular epidemiology of tuberculosis. Kekkaku.

[5] Borgdorff MW, de Haas P, Kremer K, van Soolingen D (2003). *Mycobacterium tuberculosis* Beijing genotype, the Netherlands. Emerg Infect Dis.

[6] Alzeer A, Mashlah A, Fakim N, Al-Sugair N, Al-Hedaithy M, Al-Majed S (1998). Tuberculosis is the commonest cause of pneumonia requiring hospitalization during Hajj (pilgrimage to Makkah). J Infect.

[7] Al-Hajoj SA, Zozio T, Al-Rabiah F, Mohammad V, Al-Nasser M, Sola C (2007). First insight into the population structure of *Mycobacterium tuberculosis* in Saudi Arabia. J Clin Microbiol.

[8] Kamerbeek J, Schouls L, Kolk A, van Agterveld M, van Soolingen D, Kuijper S (1997). Simultaneous detection and strain differentiation of *Mycobacterium tuberculosis* for diagnosis and epidemiology. J Clin Microbiol.

[9] Brudey K, Driscoll JR, Rigouts L, Prodinger WM, Gori A, Al-Hajoj SA (2006). *Mycobacterium tuberculosis* complex genetic diversity: Mining the fourth international spoligotyping database (SpolDB4) for classification, population genetics and epidemiology. BMC Microbiol.

[10] Kremer K, Glynn JR, Lillebaek T, Niemann S, Kurepina NE, Kreiswirth BN (2004). Definition of the Beijing/W lineage of *Mycobacterium tuberculosis* on the basis of genetic markers. J Clin Microbiol.

[11] Supply P, Allix C, Lesjean S, Cardoso-Oelemann M, Rüsch-Gerdes S, Willery E (2006). Proposal for standardization of optimized mycobacterial interspersed repetitive unit-variable-number tandem repeat typing of *Mycobacterium tuberculosis*. J Clin Microbiol.

[12] Mokrousov I, Narvskaya O, Vyazovaya A, Millet J, Otten T, Vishnevsky B (2008). *Mycobacterium tuberculosis* Beijing genotype in Russia: In search of informative variable-number tandem-repeat loci. J Clin Microbiol.

[13] Velayati AA, Farnia P, Mirsaeidi M, Reza Masjedi M (2006). The most prevalent *Mycobacterium tuberculosis* superfamilies among Iranian and Afghan TB cases. Scand J Infect Dis.

[14] Velayati AA, Masjedi MR, Farnia P, Tabarsi P, Ghanavi J, Ziazarifi AH (2009). Emergence of new forms of totally drug-resistant tuberculosis bacilli: Super extensively drug-resistant tuberculosis or totally drug-resistant strains in Iran. Chest.

[15] Farnia P, Masjedi MR, Varahram M, Mirsaeidi M, Ahmadi M, Khazampour M (2008). The recent-transmission of *Mycobacterium tuberculosis* strains among Iranian and Afghan relapse cases: A DNA-fingerprinting using RFLP and spoligotyping. BMC Infect Dis.

